# Intranasal fentanyl spray versus intravenous opioids for the treatment of severe pain in patients with cancer in the emergency department setting: A randomized controlled trial

**DOI:** 10.1371/journal.pone.0235461

**Published:** 2020-07-10

**Authors:** Srinivas R. Banala, Osama K. Khattab, Valda D. Page, Carla L. Warneke, Knox H. Todd, Sai-Ching Jim Yeung

**Affiliations:** 1 Department of Emergency Medicine, The University of Texas MD Anderson Cancer Center, Houston, Texas, United States of America; 2 Department of Emergency Medicine, Baylor College of Medicine, Houston, Texas, United States of America; 3 Department of Biostatistics, The University of Texas MD Anderson Cancer Center, Houston, Texas, United States of America; Universidad Miguel Hernandez de Elche, SPAIN

## Abstract

**Objective:**

Intranasal fentanyl (INF) quickly and noninvasively relieves severe pain, whereas intravenous hydromorphone (IVH) reliably treats severe cancer pain but requires vascular access. The trial evaluated the efficacy of INF relative to IVH for treating cancer patients with severe pain in an emergency department (ED) setting.

**Methods:**

We randomized 82 patients from a comprehensive cancer center ED to receive INF (n = 42) or IVH (n = 40). Eligible patients reported severe pain at randomization (≥7, scale: 0 “none” to 10 “worst pain”). We conducted non-inferiority comparisons (non-inferiority margin = 0.9) of pain change from treatment initiation (T0) to one hour later (T60). T0 pain ratings were unavailable; therefore, we estimated T0 pain by comparing 1) T60 ratings, assuming similar group T0 ratings; 2) pain change, estimating T0 pain = randomization ratings, and 3) pain change, with T0 pain = 10 (IVH group) or T0 pain = randomization rating (INF group).

**Results:**

At T60, the upper 90% confidence limit (CL) of the mean log-transformed pain ratings for the INF group exceeded the mean IVH group rating by 0.16 points (>pain). Substituting randomization ratings for T0 pain, the lower 90% CL of mean pain change in the INF group extended 0.32 points below (<pain relief) mean change in the IVH group. Finally, assuming all subjects in the IVH group had maximum pain at T0 and that T0 pain for the INF group remained unchanged from randomization, the lower bound of the 90% CL for mean pain decrease in the INF group extended 1.37 points below (<pain relief) mean decrease in the IVH group. Time (minutes) from randomization until T0 was longer for the IVH (*Median* 23, *IQR* 12) versus INF (*Median* 15, *IQR* 11) group (*P*<0.001).

**Conclusions:**

Two of three analyses supported non-inferiority of INF versus IVH, while one analysis was inconclusive. Compared to IVH, INF had the advantage of shorter time to administration.

**Trial registration:**

ClinicalTrials.gov Identifier: NCT02459964

## Introduction

Cancer pain is highly prevalent and adversely affects quality of life, making the treatment of pain a crucial part of oncologic care [1]. More than 50% of patients with cancer will experience physical pain, and for 30% of these, the pain will be moderate or severe [2, 3]. Given the ongoing opioid-abuse epidemic, the American Society of Clinical Oncology (ASCO) has published new guidelines for managing chronic pain in cancer survivors [4]; nonetheless, emergency physicians are shying away from prescribing opioids [5] even though pain is a significant issue for cancer patients presenting to the emergency department (ED) [6].

Most patients with chronic cancer pain experience periodic pain flare ups, or “breakthrough pain,” defined as a spontaneous or incidental pain episode of severe intensity in patients receiving otherwise adequate opioid treatment that provides at least mild analgesia. The recognition of breakthrough pain as a significant problem among patients with cancer receiving long-term opioid therapy has supported the use of the “rescue dose,” a short-acting opioid administered during breakthrough pain [7].

Intravenous (IV) hydromorphone titration (the “1+1” protocol) is a safe, well-accepted ED analgesic regimen for patients with severe pain. The regimen involves administering 1 mg of IV hydromorphone followed by an additional 1 mg if requested by the patient. The efficacy of this patient-driven 1+1 hydromorphone protocol has been found to be both clinically and statistically superior to usual care of ED patients with acute severe pain [8]. However, one barrier to using this method is that it requires assembling the materials for IV administration; in addition, establishing IV access can be difficult for some patients. Other routes of analgesia administration for expeditious pain relief would be desirable.

The intranasal (IN) route has been investigated as an alternative [9, 10], Lazanda® fentanyl nasal spray was approved by the FDA in July 2011 for the management of breakthrough pain in adult patients with cancer [11]. IN fentanyl administration might be preferred because IN delivery does not require establishment of IV access; thus, there are no inherent delays with drug-delivery preparation and administration, which would reduce time to analgesic relief compared with IV opioids [12].

For cancer patients with severe pain in the ED, ease of potent analgesic administration and time to pain relief are urgent medical needs. However, few clinical studies of analgesic therapy for cancer patients in the ED have been reported. To address this gap in the research, we conducted a randomized clinical trial to compare an IN fentanyl spray (Lazanda) to IV hydromorphone in terms of pain relief among patients with severe pain at the ED. The hypothesis was that IN fentanyl spray was not inferior to IV hydromorphone in terms of pain relief among patients who presented to the ED with severe pain.

## Methods

### Study design and setting

This was a single-center, randomized, open-label clinical trial designed to compare IN fentanyl versus IV opioids in pain intensity reduction by 1 hour after drug delivery. The Institutional Review Board of The University of Texas MD Anderson Cancer Center approved the study (Approved Protocol Number: 2015–0086). Written informed consent was obtained for all participants. Participants were adults with cancer presenting with severe pain to the ED of a comprehensive cancer center. We selected a sample size of 84 (42 per group) to have 80% power to detect non-inferiority of IN fentanyl versus IV hydromorphone using a one-sided, two-sample *t*-test, with a non-inferiority margin of 0.9 and an alpha of 0.05 [13]. For these power calculations, we based variability estimates on meta-analysis results that demonstrated unequal variability in pain change by treatment group (*SD* 1.86 IN fentanyl; *SD* 1.40 IV hydromorphone) [14]. The non-inferiority margin was chosen based on a randomized clinical trial that showed a 5.6 point pain reduction by IV hydromorphone (1 mg dose) [15], and the chosen margin of 0.9 would be 16% of that (i.e., <20% of the effect for the standard treatment) as recommended by experts [16]. Our chosen non-inferiority margin was also well below the 2-point or 30% decrease in pain scores that, per Farrar et al. [17] represents clinically significant pain relief.

Patients were enrolled in the ED between 10/13/2015 and 7/8/2016 after having the study explained to them and agreeing to participate. After providing written informed consent, participants were randomized into either the IN fentanyl arm or IV hydromorphone reference arm by simple randomization using a computer program on an institutional clinical research server.

### Selection of participants

Eligible patients had severe pain (rated ≥7 on a 0–10 numeric rating scale, where 0 = “no pain” and 10 = “pain as bad as you can imagine”) and had been on opioid therapy for 1 week or longer (at least 60 mg of oral morphine/day, 25 mcg of transdermal fentanyl/hour, 30 mg of oxycodone/day, 8 mg oral hydromorphone/day, 25 mg oral oxymorphone/day, or an equianalgesic dose of another opioid). Exclusion criteria included chronic active hepatitis, cirrhosis, or hepatic encephalopathy; known or suspected hypersensitivity or intolerance to fentanyl, hydromorphone, or excipients in the study medications; sinusitis, obstruction of nasal passages, nasopharyngeal cancer, paranasal sinus malignancies, or any conditions in the nasopharyngeal area that could affect absorption of IN fentanyl spray; having taken oral immediate-release opioids within 4 hours prior to arrival in the ED; and previous participation in this trial. In addition, women could not be pregnant, breast feeding, or intending to become pregnant; women of child-bearing potential were required to be using adequate contraceptive measures in order to participate.

### Interventions

For the intervention group, 100 mcg of fentanyl was administered at Time 0 (T0) (i.e., time of intranasal administration); for the reference group, an equipotent dose [18] of IV hydromorphone 1.5 mg was administered at T0 (defined as the time of completion of the opioid IV push). Rescue doses of the study drug were allowed at 30 minutes after T0 for patients who reported pain >4 and who desired additional analgesics at that time; however, rescue doses were not given to patients with excessive sedation (Ramsay Sedation Scale ≤4). After another 90 minutes, patients with unrelieved pain (rated >6) and Ramsay Sedation Scale >4 received supplemental IV bolus doses of opioid as clinically indicated and ordered by the treating physician.

### Measurements

We performed the measurements described in this section for 4 hours after initiation of the drug intervention, while the patient was still in the ED.

### Analysis

We summarized the study sample characteristics using simple descriptive statistics, such as the count, percentage, mean, standard deviation (SD), median, minimum, maximum, and interquartile range (IQR). Study group differences in baseline demographic and clinical characteristics were assessed using Fisher’s exact tests, Wilcoxon rank-sum tests, or independent samples *t*-tests as appropriate. Because raw pain scores were not normally distributed, the means and 90% confidence limits (CLs) for raw pain scores were constructed by taking the natural log of the scores and then exponentiating means and CLs for conversion back to the original scale. Mean change in pain scores and 90% CLs were calculated as the mean difference between the estimated pain score at T0 and the 1-hour pain score. (See below section, “Protocol Deviations and Remedies.”) Group differences in pain scores were presented as the mean change for IN fentanyl group minus that for the IV hydromorphone group. We used generalized linear methods to model the outcome (pain score or pain score decrease). We controlled for age category (dividing at the median: 22–51, > 51–84 years), race (Black, Hispanic, white, other), sex, and type of cancer (hematological, non-hematological, and non-cancer).

The prespecified non-inferiority margin was 0.9 points. Because higher raw pain scores represent worse pain, if the upper 90% CL around the mean raw pain score at 1 hour for the IN fentanyl group extended no more than 0.9 points higher than the mean pain score for the IV hydromorphone group, IN fentanyl was considered non-inferior to IV hydromorphone for pain relief. Given that a smaller decrease in pain is worse, if the lower bound of the 90% CL for the IN fentanyl group mean change in pain was no more than 0.9 points below the mean change in the IV hydromorphone group, then IN fentanyl was considered non-inferior to IV hydromorphone.

### Protocol deviation and remedies

Because of a protocol deviation, pain ratings at T0 were not collected for most patients. To statistically mitigate this omission, we applied three approaches. The first approach assumed that pain scores at T0 were equivalent for the two randomized groups and compared groups based on raw pain scores 1 hour after T0. We also compared groups on the pain categories at 1 hour after T0 (no pain = 0, mild = 1–3, moderate = 4–6, and severe = 7–10). The second approach used the pain score at randomization to estimate the pain score at T0. The third and most conservative approach (given that pain scores can increase or stay the same without treatment) assumed that all study subjects in the IV hydromorphone group had a pain score of 10 (the maximum pain score) at the start of treatment and compared the change in pain scores from 10 in the IV hydromorphone group to the change in pain scores from randomization values in the IN fentanyl group. In this scenario, any pain drop in the IV hydromorphone group would likely be overstated relative to that experienced by the IN fentanyl group. However, if the pain drop in the IN fentanyl group was not less than 0.9 points below the mean maximum possible pain drop in the IV hydromorphone group, we could be more assured that IN fentanyl was non-inferior to IV hydromorphone.

## Results

### Pre-treatment characteristics of the study participants

**Fig 1:**
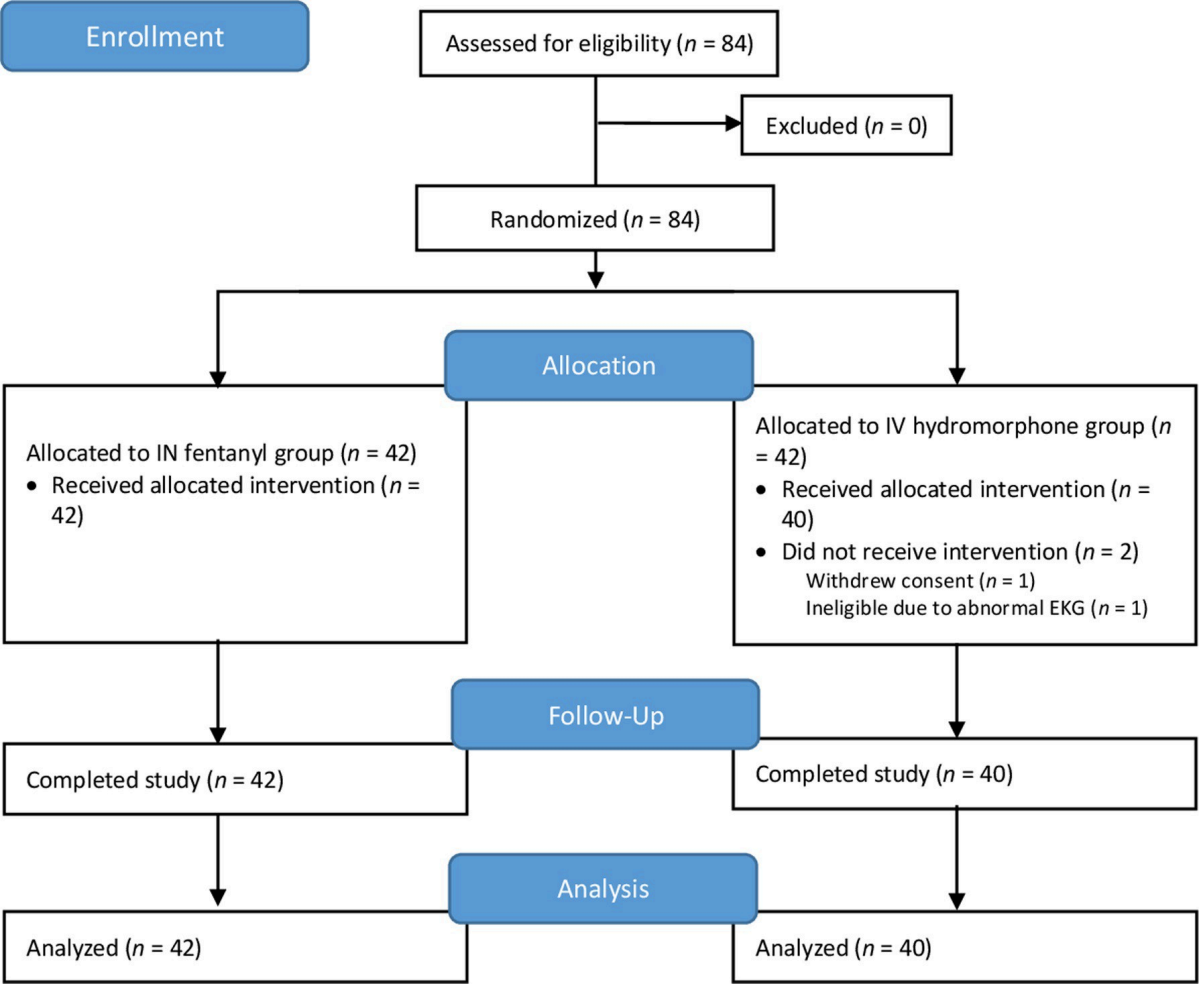
CONSORT flow diagram.

The CONSORT diagram for the study participants is shown in Fig 1, and selected patient characteristics are detailed in Table 1. The 82 evaluable participants ranged from 22–84 years of age (*Mean* 52.9 years, *standard deviation (SD)* 13.3). Most patients (79%) had solid tumors.

**Table 1:** Comparison of demographic and clinical characteristics, by treatment group.

**Variable**	**Level**	**IV Hydromorphone (*n* = 40)**				**IN Fentanyl (*n* = 42)**				**Total**		***P*** *
		***n***		**%**		***N***		**%**		***n***	**%**	
Sex	Female	23		57.5		23		54.8		46	56.1	0.83
	Male	17		42.5		19		45.2		36	43.9	
Race	White	29		72.5		20		47.6		49	59.8	0.08
	Hispanic	6		15.0		10		23.8		16	19.5	
	Black	3		7.5		10		23.8		13	15.9	
	Other	2		5.0		2		4.8		4	4.9	
Type of cancer diagnosis	Non-hematological	33		82.5		33		78.6		66	80.5	0.89
	Hematological	6		15.0		8		19.0		14	17.1	
	Non-cancer	1		2.5		1		2.4		2	2.4	
		**IV Hydromorphone**				**IN Fentanyl**				**Total *n***		***P*** *
		**Median**	**Min**		**Max**	**Median**	**Min**		**Max**			
Age at ED visit, years		51	29		78	55	22		84	82		0.17
Minutes from arrival to triage		7	0		30	9.5	1		26	82		0.10
Minutes from triage to randomization		37	12		135	37	16		166	82		0.74
Minutes from arrival to randomization		48	26		142	48	22		178	82		0.43
Minutes from randomization to treatment initiation		23	6		83	15	4		67	82		<0.001
Pain rating at triage		8	4		10	8.5	2		10	73		0.82
Pain rating at randomization		9	7		10	8.5	7		10	82		0.28

* *P*-values are from the Fisher’s exact test for categorical variables and the Wilcoxon rank-sum test for quantitative variables.

Abbreviations: ED, emergency department; IN, intranasal; IV, intravenous.

The two treatment groups did not differ significantly by age, sex, race, type of cancer diagnosis, pain rating at triage, pain rating at randomization, time from arrival to triage, time from triage to randomization, or time from arrival to randomization (Table 1).

The time from ED arrival to randomization varied from a minimum of 22 to a maximum of 178 minutes (*Median* 47.5, *IQR* 34), and the time from triage to randomization ranged from 12 to 166 minutes (*Median* 37, *IQR* 28). At the time of randomization, the pain scores, per eligibility criteria, spanned from 7 to 10 with a median score of 9.

**Fig 2:**
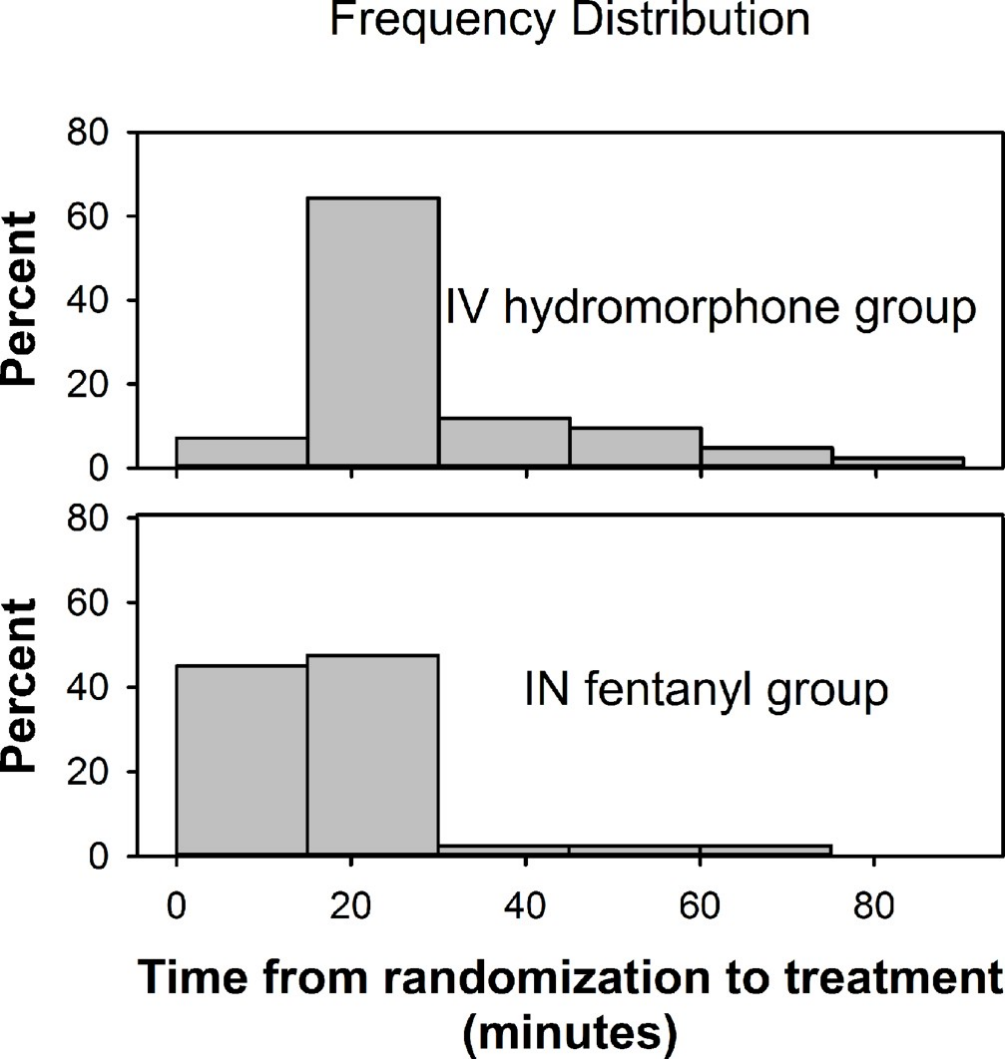
Time from randomization to treatment initiation (T0), by treatment group. Abbreviations: IN, intranasal; IV, intravenous.

The time from randomization to T0 varied from 4 to 83 minutes with a median time of 19 minutes (*IQR* 12). Time from randomization to T0 was significantly shorter for the IN fentanyl group (*Median* 15 minutes, *IQR* 11) than for the IV hydromorphone group (*Median* 23 minutes, *IQR* 12) (*P* <0.001) (Fig 2).

### Pain scores 1 hour after treatment initiation (T0)

**Fig 3:**
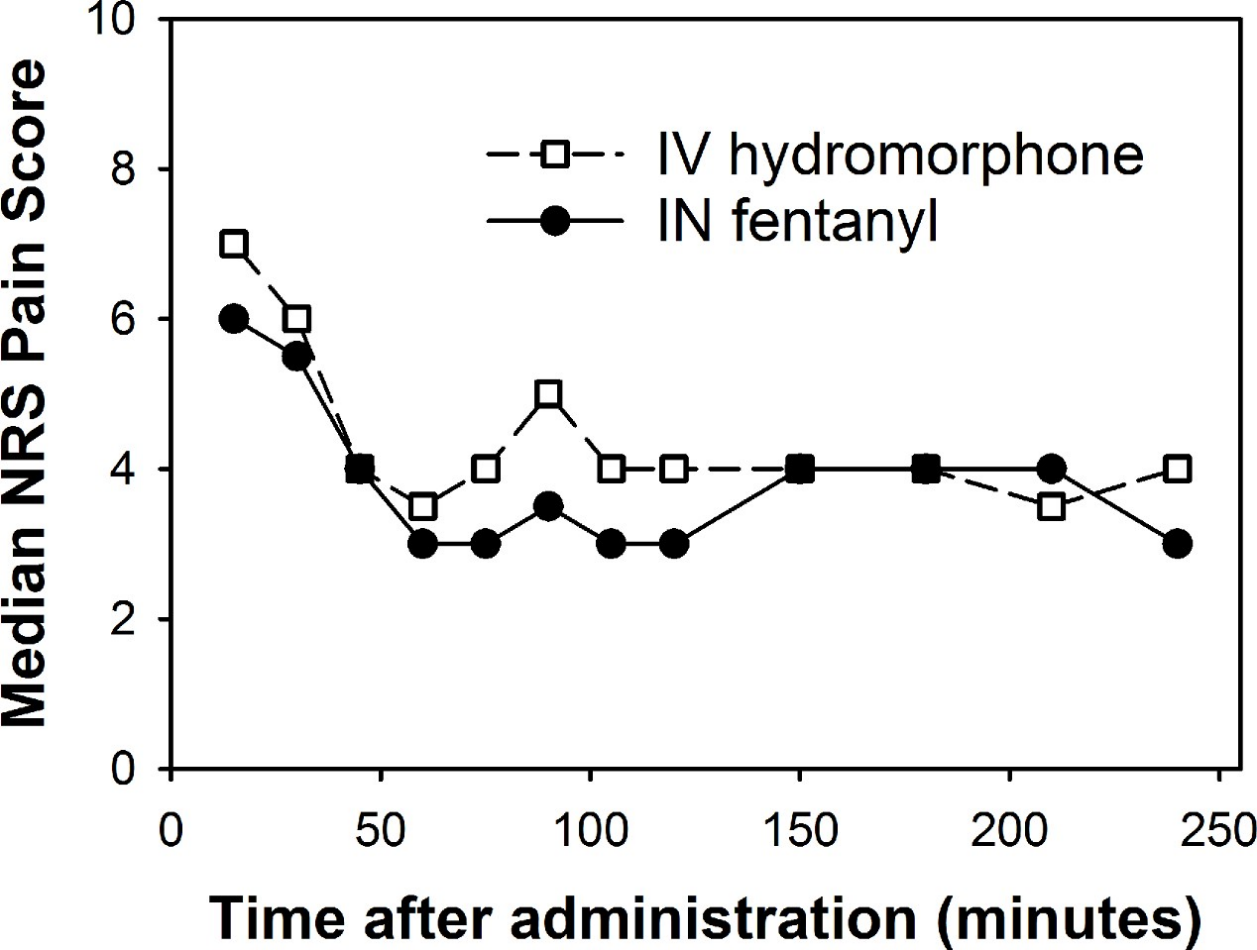
Median pain scores across time, by treatment group. Abbreviations: IN, intranasal; IV, intravenous; NRS, numerical rating scale.

A plot of median pain scores across time shows that the most dramatic drop in pain occurred within 45–60 minutes after T0 and roughly leveled off afterwards (Fig 3). The median pain score at randomization was 8.5 for the IN fentanyl group and 9 for the IV hydromorphone group. The group difference in pain scores at randomization was not statistically significant (Fisher’s exact test, *P* = 0.67) (S1 Table).

One hour after T0, pain scores ranged from 0 to 8 with a median of 3.5 (*IQR* 4) in the IV hydromorphone group and from 0 to 10 with a median of 3 (*IQR* 3) in the IN fentanyl group (Table 2). For the IN fentanyl group, the upper bound of the 90% CL of the mean pain score 1 hour after T0 was 0.16 points higher (i.e., more pain) than the mean score for the IV hydromorphone group. This was within the 0.9-point non-inferiority margin.

**Table 2:** Pain ratings 60 minutes after treatment initiation (T0), by treatment group.

	Treatment Group	
	IV Hydromorphone (*n* = 40)	IN Fentanyl (*n* = 42)
Percent severe (7–10)	25%	12%
Median score (IQR)	3.5 (4.0)	3.0 (3.0)
Mean (SD) decrease from pain score at randomization	4.90 (2.31)	5.14 (2.16)
Mean (SD) decrease from maximum pain score of 10 for the IV hydromorphone group and from randomization pain score for the IN fentanyl group	5.95 (2.39)	5.14 (2.16)

Abbreviations: ED, emergency department; IN, intranasal; IQR, interquartile range; IV, intravenous; SD, standard deviation.

In addition to comparing groups on raw scores at one hour T0, we compared groups on pain score categories. One hour after T0, 12% of participants reported no pain (rating 0), 41% mild pain (rating 1–3), 28% moderate pain (rating 4–6), and 18% severe pain (rating 7–10). Score categories did not differ significantly by treatment group (Fisher’s exact test *P* = 0.49) (S2 Table). The percentage of participants with continued severe pain at 1 hour after T0 was 25% in the IV hydromorphone group and 12% in the IN fentanyl (12%) group.

### Pain score change from treatment initiation (T0) to 1 hour later

#### Estimating pain at T0 as the pain rating at randomization

**Fig 4:**
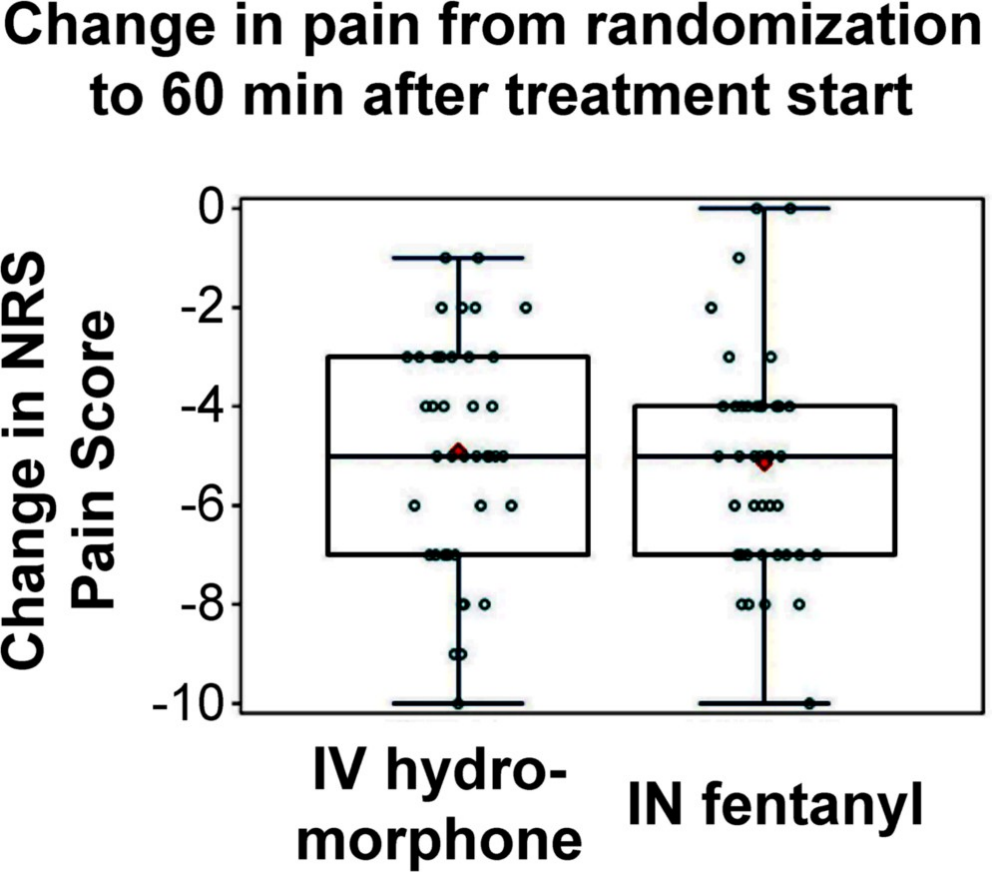
Change in pain scores from randomization to 60 minutes after treatment initiation (T0), by treatment group. Abbreviations: IN, intranasal; IV, intravenous; NRS, numerical rating scale. Individual points are jittered for readability.

Using the pain score at randomization as an estimate of the pain score at T0, mean pain scores dropped by 5.02 points (*SD* 2.22) at 1 hour after treatment, ranging from no change to a drop of 10 points. Scores decreased an average of 5.14 points (*SD* 2.16) for the IN fentanyl group and 4.90 points (*SD* 2.31) for the IV hydromorphone group (Fig 4 and Table 3). One hour after treatment, all but 2 participants had a decrease in pain scores. The 2 participants reporting no decrease in pain were both in the IN fentanyl group and had pain scores of 9 and 10 at randomization.

**Table 3:** Decrease in pain ratings from treatment initiation (T0) to one hour later, using two methods to estimate pain ratings at T0.

T0 Pain Score Estimation Method	IV Hydromorphone *n* = 40		IN Fentanyl *n* = 42		Difference (IN Fentanyl–IV Hydromorphone)		*Margin
	Mean	SD	Mean	SD	Mean	SD	
Carry forward pain score at randomization for both groups	4.90	(2.31)	5.14	(2.16)	0.24	(2.23)	0.32
Assume maximum pain score (10) for the IV hydromorphone group and carry forward randomization pain scores for the IN Fentanyl group	5.95	(2.39)	5.14	(2.16)	–0.81	(2.27)	1.37

*Margin: Mean pain decrease in IV Hydromorphone group minus lower 90% confidence limit for mean decrease in IN Fentanyl

Lower scores indicate better pain relief. Pain scores at treatment initiation were unavailable.

Abbreviations: CL, confidence limit; IN, intranasal; IV, intravenous; SD, standard deviation.

From randomization to 1 hour after T0, the average IN fentanyl pain score dropped 0.24 points (*SD* 2.23) more than the average pain decrease in the IV hydromorphone group (Table 3). The lower bound of the 90% CL for the mean decrease in pain scores for the IN fentanyl group extended to 0.32 points below (less pain relief) the mean change in the IV hydromorphone group. This was within the prespecified non-inferiority margin of 0.90 points, supporting the non-inferiority of IN fentanyl.

#### Estimating pain at T0 as maximum possible pain in the IV hydromorphone group

Pain scores at randomization may not have represented a valid estimate of pain at T0, especially for the IV hydromorphone group, which had a significantly longer time lapse between randomization and T0. For the IN fentanyl group, the median time lapse was 15 minutes (*IQR* 11) versus a median lapse of 23 minutes (*IQR* 12) for the IV hydromorphone group (Wilcoxon rank-sum test *P*<0.001). At randomization, 55% of the IV hydromorphone group rated pain at 7–9 and 45% rated pain at 10. If the IV hydromorphone participants were assumed to have a T0 pain score that increased to 10 during the time lapse and the IN fentanyl participants were assumed to have a T0 score equal to the pain score at randomization, the mean decrease in pain scores was 5.95 (*SD* 2.39) points for the IV hydromorphone group and 5.14 (*SD* 2.16) points for the IN fentanyl group. One hour after T0, the mean pain relief was less for the IN fentanyl group than for the IV hydromorphone group by 0.81 points (*SD* 2.27) (Table 3). The lower bound of the 90% CL for the IN fentanyl group decrease in pain extended 1.37 points lower (less pain relief) than the mean decrease in the IV hydromorphone group, meaning that the non-inferiority of IN fentanyl to IV hydromorphone cannot be concluded in this scenario based on the non-inferiority margin of 0.9 points.

### Multivariable analyses

Multivariable models for each approach described in the section, Protocol Deviations and Remedies, included age, race, sex, and cancer type as covariates. For the first approach (assuming that pain scores at T0 were equivalent for the two randomized groups [IN fentanyl (INF) vs. IV hydromorphone (IVH)], and comparing groups based on raw pain scores 1 hour after T0), the margin went from 0.16 to 0.45 points higher (more pain) based on the INF group’s upper 90% confidence limit (CL) versus the IVH group’s mean pain score. For the second approach (using the pain score at randomization to estimate the pain score at T0), the margin changed from 0.32 to 0.85 points less pain relief for the INF versus IVH group. For the third approach (assuming maximum possible pain score change for only the IVH group), the margin went from 1.37 to 1.88 points less pain relief for the INF versus IVH group. The mean pain score decreased 5.4 (95% CI: 4.5, 6.3) points for the INF group and 6.3 (95% CI: 5.4, 7.2) points for the IVH group when controlling for age, race, sex, and cancer type. Therefore, the multivariable models using approaches 1 and 2 supported that INF was non-inferior to IVH, but the model per approach 3 did not meet our preset criterion for non-inferiority.

## Limitations

The major limitation of this study was a protocol deviation that led to missing pain scores at the start of treatment (T0). Other weaknesses were the lack of blinding, the subjectivity of self-reported pain ratings, and conducting the study at a single institution. A limitation is the use of time of administration instead of time of decision for the data analysis, and our results should be interpreted with caution when applied to real life clinical practice outside the clinical research setting. Another limitation was the small sample size of this study. Although our sample size was adequate for assessing non-inferiority using a 0.9-point non-inferiority margin, we would not be able to assess equivalence with this margin unless the sample size was almost doubled. Also, a larger sample size would be needed if the non-inferiority margin is to be reduced.

## Discussion

As the first randomized, open-label study to compare administration of IN and IV opioids to treat acute pain episodes among patients with cancer in an ED setting, this study demonstrated that the use of noninvasive IN fentanyl can expeditiously address the problem of severe cancer pain in the ED. One of our important findings was that IN fentanyl can be given to the patients faster than can IV hydromorphone.

IN fentanyl has been shown to be comparable to IV opioids for management of a variety of analgesic indications [19], but we are not aware of any studies directly assessing non-inferiority of IN fentanyl to IV opioids in adults. In a blinded, randomized, placebo-controlled study [20], the efficacy of IV morphine and IN fentanyl in children/adolescents with long bone fractures in an emergency center found no difference between IV morphine and IN fentanyl in pain reduction. A subsequent review of published studies in children found insufficient evidence to make any definitive conclusions about non-inferiority or equivalency of IN fentanyl to IV or IM morphine [21].

Advantages to an IN mode of fentanyl administration include a noninvasive route of administration, rapid onset of action, high bioavailability with avoidance of hepatic first-pass metabolism, and high patient acceptability. Compared to oral transmucosal or buccal forms of fentanyl, IN fentanyl may be used in patients with radiation-induced dry mouth. In addition, a recent meta-analysis reported that IN fentanyl has a faster onset of action than oral transmucosal fentanyl citrate, buccal fentanyl tablets, or oral morphine [22]. However, in real-life ED workflows, the biggest time delay in pain relief after a physician recognizes the patient’s need is often the time needed to get the pain medication ready for administration; for IV drugs, this will include establishing IV access, which may be problematic and time consuming for a fair portion of patients.

We found that the time between randomization and starting treatment (T0) was significantly shorter in the IN fentanyl group than the IV hydromorphone group. In the clinical trial setting, the study medications were controlled by the research pharmacy, but in routine clinical practice, the time from physician order to IN fentanyl administration can be much shorter if this medication is stocked in an automated medication dispensing system. Even in a simulation classroom setting, IN administration of medication is faster than IV medication to a mannequin model, taking about 90 more seconds for the IV route than IN [23]. For cancer patients in real-life clinical practice, difficulty with establishment of IV access is a frequent problem, requiring extraordinary effort and expertise to address.

The raw pain scores 1 hour T0 suggested that IN fentanyl was not inferior to IV hydromorphone. However, the primary objective of this study was to test the non-inferiority of IN fentanyl versus IV hydromorphone in the *change* in pain intensity after 1 hour from the time of drug delivery (T0) in adult patients presenting to the ED with severe pain. But a protocol deviation led to missing pain scores at the start of treatment (T0).

In an attempt to address this limitation, three different strategies were applied: assuming similar T0 pain scores in the randomized groups and comparing raw pain scores at one hour after T0; using pain scores at randomization to estimate T0 pain; and assuming maximum pain at T0 for the IV hydromorphone group. With the first approach, pain scores in the IN fentanyl group were no more than 0.2 points higher than in the IV hydromorphone group, which was within the 0.9-point non-inferiority margin. The second approach carried forward pain scores at randomization, in effect assuming no change in pain scores after randomization until T0. Again, results indicated that the pain relief of IN fentanyl was non-inferior to that of IV hydromorphone. However, the assumption of no significant change in pain scores between randomization and T0 may not be valid due to the significantly longer time lapse in the IV hydromorphone group. Our third approach attempted to account for study group differences in the time-lapse by assuming a maximum pain score for the IV hydromorphone group. With this strategy, if the pain relief of IN fentanyl was non-inferior to IV hydromorphone, we could safely assume that IN fentanyl was non-inferior. However, the results of this strategy failed to show that IN fentanyl was non-inferior to IV hydromorphone, given the prespecified non-inferiority margin of 0.9 points. Therefore, results based on the prespecified non-inferiority margin were inconclusive. Overall, our results suggest that pain relief 1 hour after T0 could be as much as 1.4 points less effective for IN fentanyl than for IV hydromorphone.

In conclusion, this randomized, open-label clinical trial found that IN fentanyl versus IV hydromorphone was administered more quickly to patients with cancer in the ED who had severe pain. Not having to wait for establishment of IV access is an advantage for prompt pain relief. Although results from our most conservative approach suggested that pain relief 1 hour after treatment could be 1.4 points less effective for IN fentanyl than for IV hydromorphone, IN fentanyl 100 mcg is very likely non-inferior to IV hydromorphone 1.5 mg. Future research will be needed to confirm our findings in a multicenter setting.

## Supporting information

S1 Table Pain ratings at randomization, by treatment group. (DOCX)

S2 Table Pain category 60 minutes after treatment initiation (T0), by treatment group. (DOCX)

S1 File CONSORT 2010 checklist of information to include when reporting a randomised trial*. (DOC)

S2 File (PDF)

S3 File (DOCX)

S4 File (PDF)

S5 File (CSV)
