# Supplementary material for: Intranasal fentanyl spray versus intravenous opioids for the treatment of severe pain in patients with cancer in the emergency department setting: A randomized controlled trial
Source: PLoS One. 2020 Jul 10;15(7):e0235461. doi: 10.1371/journal.pone.0235461 (PMC7351205; doi:10.1371/journal.pone.0235461)
Supplement: S1 Table — (DOCX) [file pone.0235461.s001.docx]

**S1 Table. Pain Ratings at Randomization, by Treatment Group.**

| \| \|  \| **Treatment Group** \| \| \| \| --- \| --- \| --- \| --- \| \| **IV Hydromorphone** \| **IN Fentanyl** \| **Total** \| \| **Pain Rating** \| ***n* (Expected *n*)** \| ***n* (Expected *n*)** \| \| 7 \| 5 (5.4) \| 6 (5.6) \| 11 \| \| 8 \| 10 (12.2) \| 15 (12.8) \| 25 \| \| 9 \| 7 (6.8) \| 7 (7.2) \| 14 \| \| 10 \| 18 (15.6) \| 14 (16.4) \| 32 \| \| Total \| 40 \| 42 \| 82 \| \| \| --- \| --- \| --- \| --- \| --- \| --- \| --- \| --- \| --- \| --- \| --- \| --- \| --- \| --- \| --- \| --- \| --- \| --- \| --- \| --- \| --- \| --- \| --- \| --- \| --- \| --- \| --- \| --- \| --- \| --- \| --- \| |  |
| --- | --- | --- | --- | --- | --- | --- | --- | --- | --- | --- | --- | --- | --- | --- | --- | --- | --- | --- | --- | --- | --- | --- | --- | --- | --- | --- | --- | --- | --- | --- | --- | --- |

Abbreviations: IN, intranasal; IV, intravenous.

Expected *n* represents the expected cell frequency under the null hypothesis of no association given the marginal distribution of the contingency table. Fisher’s exact test *P* = 0.67
