# Supplementary material for: Intranasal fentanyl spray versus intravenous opioids for the treatment of severe pain in patients with cancer in the emergency department setting: A randomized controlled trial
Source: PLoS One. 2020 Jul 10;15(7):e0235461. doi: 10.1371/journal.pone.0235461 (PMC7351205; doi:10.1371/journal.pone.0235461)
Supplement: S2 Table — (DOCX) [file pone.0235461.s002.docx]

#### S2 Table. Pain Category 60 Minutes after Treatment Initiation (T0), by Treatment Group

|  | **Treatment Group** | | |
| --- | --- | --- | --- |
|  | **IV Hydromorphone** | **IN Fentanyl** | **Total** |
| **Pain Rating Category** | ***n* (%)** | ***n* (%)** |  |
| No pain (0) | 4 (10.0) | 6 (14.3) | 10 |
| Mild pain (1, 2, 3) | 16 (40.0) | 18 (42.9) | 34 |
| Moderate pain (4, 5, 6) | 10 (25.0) | 13 (31.0) | 23 |
| Severe pain (7, 8, 9, 10) | 10 (25.0) | 5 (11.9) | 15 |
| Total | 40 | 42 | 82 |

Abbreviations: IN, intranasal; IV, intravenous
