# Supplementary material for: Intranasal fentanyl spray versus intravenous opioids for the treatment of severe pain in patients with cancer in the emergency department setting: A randomized controlled trial
Source: PLoS One. 2020 Jul 10;15(7):e0235461. doi: 10.1371/journal.pone.0235461 (PMC7351205; doi:10.1371/journal.pone.0235461)
Supplement: S2 File — (PDF) [file pone.0235461.s004.pdf]

THE UNIVERSITY OF TEXAS
MD Anderson ~~Cancer~~ Center

Protocol Page

A randomized trial to compare fentanyl nasal spray with intravenous opioids to treat severe pain in cancer patients in the emergency department setting.
2015-0086

Core Protocol Information

**Short Title**	Compare fentanyl nasal spray with intravenous opioids to treat severe pain
**Study Chair:**	Sai-Ching J. Yeung
**Additional Contact:**	Denise M. Langabeer<br>Jessica A. Nollkamper<br>Valda Page
**Department:**	Emergency Medicine
**Phone:**	713-745-9911
**Unit:**	1468
**Full Title:**	A randomized trial to compare fentanyl nasal spray with intravenous opioids to treat severe pain in cancer patients in the emergency department setting.
**Protocol Type:**	Standard Protocol
**Protocol Phase:**	N/A
**Version Status:**	Activated -- Closed to new patient entry as of 08/15/2018
**Version:**	08
**Submitted by:**	Sai-Ching J. Yeung--8/15/2018 11:43:00 AM
**OPR Action:**	Accepted by: Inar Y. Graur -- 8/15/2018 4:00:23 PM

Which Committee will review this protocol?

(•) The Clinical Research Committee - (CRC)

Protocol Body

Lazanda RCT 03202015.docx Departmental Prioritization List.xlsx
