# Supplementary material for: Intranasal fentanyl spray versus intravenous opioids for the treatment of severe pain in patients with cancer in the emergency department setting: A randomized controlled trial
Source: PLoS One. 2020 Jul 10;15(7):e0235461. doi: 10.1371/journal.pone.0235461 (PMC7351205; doi:10.1371/journal.pone.0235461)
Supplement: S3 File — (DOCX) [file pone.0235461.s005.docx]

**Title:** A randomized trial to compare fentanyl nasal spray with intravenous opioids to treat severe pain in cancer patients in the emergency department setting.

**Study Chair:**

Sai-Ching Jim Yeung

**Study Co-Chair**

Knox Todd

**1. Objectives**

The **primary objective** of this project is to test the non-inferiority of fentanyl nasal spray versus intravenous opioids in the change in pain intensity at one hour, starting from the time of drug delivery, in adults with cancer presenting to the ED with severe pain.

**Secondary Objectives:**

1. To compare the change in pain intensity [measured using an 11-point (0 to 10) Numeric Rating Scale (NRS)] at one hour after randomization in the fentanyl nasal spray arm to that in the intravenous opioid arm among adults with cancer presenting to the Emergency Department (ED) with severe pain (NRS≥7).
2. To monitor the safety and side effects of fentanyl nasal spray and intravenous opioids in adults with cancer presenting to the ED with severe pain.
3. To estimate the time from randomization to IV establishment in adults with cancer presenting to the ED with severe pain.

**Exploratory Objectives:**

1. To compare the time to maximal pain relief of fentanyl nasal spray and intravenous opioids in adults with cancer presenting to the ED with severe pain.
2. To compare the summed pain intensity difference of fentanyl nasal spray and intravenous opioids in adults with cancer presenting to the ED with severe pain.

**2. Background**

It is estimated that approximately 30 to 50% of patients undergoing antineoplastic therapy and 75 to 90% of patients with advanced cancer have chronic pain severe enough to warrant opioid therapy (Kanner, 1996; Vainio A, 1996; WHO, 1996). In 18 studies reporting pain severity among cancer patients, one-third of patients rated pain as moderate to severe (Everdingen, 2007). Unfortunately, many of these patients are under-treated. Recent studies performed both in Italy and in Europe demonstrated that pain was present in all phases of the cancer spectrum and was not adequately treated in a large number of patients, ranging from 56% to 82% (Constantini, 2009; Breivik, 2009).

Acute breakthrough cancer pain is commonly caused by diagnostic or therapeutic interventions such as surgery (e.g., pneumonectomy for lung cancer). Chronic cancer pain is more associated with cancer itself or to antineoplastic therapy. Most patients with chronic cancer pain will also experience periodic flare ups of pain, or “breakthrough pain” (Fine, 2003). The recognition of breakthrough pain as a significant problem among cancer patients in the midst of their long-term opioid therapy has supported the use of “rescue doses”. The rescue dose is often a short-acting opioid administered during the midst of cancer breakthrough pain (Portenoy, 2009). All patients who are receiving slow-release opioids should also have access to immediate-release opioids that can be used for breakthrough pain (Bruera, 2003).

Pain is one of the leading reasons for seeking care in the ED (Cordell, 2002), yet it is often undertreated in the ED setting because of inadequate assessment and treatment or fears of addiction (Rupp, 2004). In addition, there are few well-designed clinical studies of analgesic therapy in the ED setting (Todd, 2004).

Two recently published reports conclude that pain management practices in emergency departments have much room for improvement. The term, “oligoanalgesia”, has been coined to describe the underutilization of analgesics in the emergency room setting. Ritsema et al. (2007) reported a study of national scope examining the quality of emergency department pain management for long-bone fractures. According to the report only 50% of subjects received a dose of an opioid analgesic for pain relief. Similarly, Todd et al. (2007) reported a multicenter emergency department study in which median pain intensity upon arrival was rated as severe, or at least 8 out of 10, on an 11-point scale (from 0 to 10). Only 60% of subjects received any analgesic, with half of those treated receiving an opioid or opioid/NSAID, and even then after a median delay of 90 minutes for administration. They ascribe these results to overcrowded facilities and physicians focusing more on the cause of the symptoms than treating the pain. A paper by Fry and Holdgate (2002) suggests that there could be greater use of nurse-initiated analgesic administration under physician-directed protocol to improve the standards and quality of care. One could speculate that improved treatment of acute pain will reduce the incidence of “central sensitization” from post-traumatic injury pain, and subsequent transition of an acute pain incident into a chronic pain condition, with all the morbidity and dysfunction subsequent to this outcome.

Intravenous hydromorphone titration (the “1+1” protocol) is a well-accepted ED analgesic regiment to allow safe analgesia to patients presenting with severe pain. The regimen involves administering 1 mg of hydromorphone intravenously, followed by an additional 1 mg if requested by the patient. The efficacy of the “1+1” hydromorphone patient-driven protocol has been found both clinical and statistically superior to usual care of ED patients with acute severe pain. (Chang, 2011)

One barrier to delivering effective ED analgesia for severe pain using this method is that it requires assembling the materials for intravenous (IV) administration. Particularly if this is the only reason to establish IV access, other routes of analgesic administration would be useful. Kendall et al. (2001) addressed this problem by conducting a study of nasal diamorphine (the chemical cousin of hydromorphone) as compared to intramuscular morphine in children and teenagers with long-bone fractures. The study demonstrated rapid onset of pain relief that was superior to intramuscular morphine and that subjects much preferred the nasal spray to an injection. They concluded that there was no longer a need to administer intramuscular morphine given this data.

Gahir and Ransom (2006) have developed an integrated care pathway involving intranasal diamorphine for pediatric analgesia in an accident and emergency department. The standard of care protocol for use of intranasal diamorphine provided clear directions for nursing staff to intervene quickly with the nasal spray analgesic. Borland et al. (2007) has also reported a study of intranasal fentanyl compared to IV morphine using a double-dummy design in long-bone fracture subjects presenting to the emergency department. They report that nasal fentanyl and IV morphine at standard doses provide equivalent rates and depths of analgesia. They conclude that nasal fentanyl administration may be preferred since there are no inherent delays with drug delivery preparation and administration and consequently nursing staff are able to “reduce time to analgesia”.

***The sum of experience from these studies is that there remains a significant unmet medical need to provide prompt analgesia in emergency departments. Creative clinical scientists have attempted to use drug delivery, and specifically nasal drug delivery, to address the issues of time to drug administration and to analgesia.*** More importantly, in the Emergency Department (ED) setting, the ease of analgesic administration and time to pain relief are of primary concern. Because intranasal delivery does not require establishment of intravenous access, intranasal fentanyl spray may potentially provide pain relief more rapidly than intravenous opioids. We propose to compare fentanyl nasal spray (Lazanda) to intravenous opioids in terms of how well pain relief can be achieved among ED patients of a comprehensive cancer center who present with severe pain.

**3 Rationale for Agent Selection**

Lazanda (fentanyl) nasal spray has been approved by the FDA (July 2011) for the management of breakthrough pain in adult cancer patients. The drug has been approved for persistent cancer pain among patients already receiving and tolerant to opioid therapy. Intranasal fentanyl provides a new approach to managing the acute severe pain that many patients with cancer experience.

Advantages to an intranasal mode of fentanyl administration include a rapid onset of action, a noninvasive route of administration, high bioavailability with avoidance of hepatic first-pass metabolism and high patient acceptability. As compared to other rapid acting forms of fentanyl (oral transmucosal or buccal fentanyl) intranasal fentanyl may be used in the patients with radiation-induced xerostomia (dry mouth). In addition, a recent meta-analysis reports that intranasal fentanyl has a faster onset of action than oral transmucosal fentanyl citrate, buccal fentanyl tablets, or oral morphine (Vissers, 2010).

**4. Study Drug Information**

**Description**

Lazanda (fentanyl) nasal spray is a liquid formulation of fentanyl citrate intended for intranasal transmucosal administration. The product consists of a clear, colorless, aqueous solution of fentanyl citrate in a glass multidose container to which is attached a metered-dose nasal spray pump with a visual and audible spray counter. Each actuation is designed to deliver a spray of 100 mcL of solution containing 100 mcg fentanyl base. This enables doses of 100 mcg to be administered using a single spray into one nostril (1 spray).

**Active ingredient**: Fentanyl citrate, USP is N-(l-phenethyl-4-piperidyl) propionanilide citrate (1:1). Fentanyl is a highly lipophilic compound (octanol-water partition coefficient at pH 7.4 is 816:1). Fentanyl citrate is sparingly soluble in water (1:40). The molecular weight of the free base and citrate salt are 336.5 and 528.6, respectively. The pKa is 8.4.

Lazanda is available in 2 strengths of nasal spray: 100 mcg fentanyl (yellow label) and 400 mcg fentanyl (violet label). The strength is expressed as the amount of fentanyl free base per spray, e.g., the 100 mcg strength provides 100 mcg of fentanyl free base per 100 mcL spray.

**Inactive ingredients**: mannitol, pectin, phenylethyl alcohol, propylparaben, sucrose, water. Sodium hydroxide and/or hydrochloric acid are added if required for pH adjustment.

**Mechanism of Action**

Fentanyl is a pure opioid agonist whose principal therapeutic action is analgesia. Other members of the class known as opioid agonists include substances such as morphine, oxycodone, hydromorphone, codeine, and hydrocodone.

**Pharmacokinetics**

*Absorption****:***

In a study that compared the relative bioavailability of Lazanda and an oral transmucosal fentanyl citrate product, the bioavailability of fentanyl from Lazanda was approximately 20% higher. Fentanyl is absorbed from the nasal mucosa following intranasal administration of Lazanda, with median Tmax values ranging from 15-21 min after administration of a single dose. Cmax and AUC values for fentanyl following administration of Lazanda increase linearly over the 100- to 800-mcg dose range.

*Distribution****:***

Fentanyl is highly lipophilic. The plasma protein binding of fentanyl is 80% to 85%. The main binding protein is alpha-1-acid glycoprotein, but both albumin and lipoproteins contribute to some extent. The mean volume of distribution at steady state (Vss) was 4 L/kg.

*Metabolism****:***

The metabolic pathways following intranasal administration of Lazanda have not been characterized in clinical studies. The progressive decline of fentanyl plasma concentrations results from the uptake of fentanyl in the tissues and biotransformation in the liver. Fentanyl is metabolized in the liver and in the intestinal mucosa to norfentanyl by cytochrome P450 3A4 isoform. In animal studies, norfentanyl was not found to be pharmacologically active.

*Elimination****:***

The disposition of fentanyl following intranasal administration of Lazanda has not been characterized in a mass balance study. Fentanyl is primarily (more than 90%) eliminated by biotransformation to N-dealkylated and hydroxylated inactive metabolites. Less than 7% of the administered dose is excreted unchanged in the urine, and only about 1% is excreted unchanged in the feces. The metabolites are mainly excreted in the urine, while fecal excretion is less important. The total plasma clearance of fentanyl following intravenous administration is approximately 42 L/h.

**Adverse Reactions**

**Clinical Studies Experience**

Because clinical trials are conducted under widely varying conditions, adverse event rates observed in the clinical trials of a drug product cannot be directly compared to rates in the clinical trials of another drug and may not reflect the rates observed in practice. The safety of Lazanda has been evaluated in a total of 523 opioid-tolerant patients with breakthrough cancer pain. The average duration of therapy in patients in the long-term study was 73 days, with 153 patients being treated for over 3 months. Patients continuing into the open-label extension period of the safety study have been treated for up to 26 months. The most commonly observed adverse events seen with Lazanda are typical of opioid side effects, such as nausea, constipation, somnolence, and headache.

**5 Eligibility Criteria**

The specific patient population to be studied is the patients presenting to the emergency center for treatment of severe pain in the context of cancer-related pain.

**Inclusion Criteria:**

1. Cancer patients with severe pain (i.e., ≥7 on NRS, see Table 1) already on opioid therapy for one week or longer, at least 60 mg of oral morphine/day, 25 mcg of transdermal fentanyl/hour, 30 mg of oxycodone/day, 8 mg oral hydromorphone/day, 25 mg oral oxymorphone/day, or an equianalgesic dose of another opioid.
2. Ability to give informed consent before any trial-related activities (Trial-related activities are any procedure that would not have been performed during normal management of the subject.)
3. Ability and willingness to communicate the intensity of pain using NRS at the frequency dictated by the protocol

**Exclusion Criteria:**

1. Patients with a history of chronic active hepatitis, cirrhosis or hepatic encephalopathy
2. Inability to give informed consent
3. Known or suspected hypersensitivity or intolerance to fentanyl or hydromorphone or excipients in the study medications
4. Patients with sinusitis, obstruction of nasal passages, nasopharyngeal cancer, paranasal sinus malignancies, or any conditions in the nasopharyngeal anatomical area that may affect the absorption of fentanyl nasal spray.
5. Females who are pregnant, breast-feeding or intending to become pregnant
6. Females of child-bearing potential, who are not using adequate contraceptive measures (including condoms, birth control pills, intrauterine devices, contraceptive implants, or other US FDA-approved contraceptives)
7. Previous participation in randomization in this trial
8. Has taken oral immediate release opioids within 4 hours prior to arrival.

Table 1. Numeric Rating Scale (NRS)

| **Rating** | **Pain Level** |
| --- | --- |
| 0 | No Pain |
| 1 – 3 | Mild Pain (nagging, annoying, interfering little with [ADLs](http://en.wikipedia.org/wiki/Activities_of_daily_living)) |
| 4 – 6 | Moderate Pain (interferes significantly with ADLs) |
| 7 – 10 | Severe Pain (disabling; unable to perform ADLs) |

*ADLs – Activities of daily living

**6 Patient Recruitment and Informed Consent**

When a cancer patient presents to the ED for treatment of severe pain, we will assess their severity of pain using an 11-point (0 to 10) NRS (Table 1) at triage. At the ED, eligible cancer patients will be approached for informed consent to participate in this study in the Triage Area immediately after ascertainment of the complaint of severe pain. If the NRS score is 7 or higher, the research nurse will conduct a brief screening physical examination (including vital signs and nasal exam), and review their medical history for eligibility (Figure A). If we determine that the patient is eligible for the study, the research nurse will initiate a formal, informed consent process, discussing the risks, benefits, and study procedures with the patient, and answer any questions the patient has to the best of his/her ability. The patient will be given his/her own personal copy of the written, informed consent document. This process will be conducted while the patient is waiting for room assignment in the ED.

**7 Treatment Plan (Figure A)**


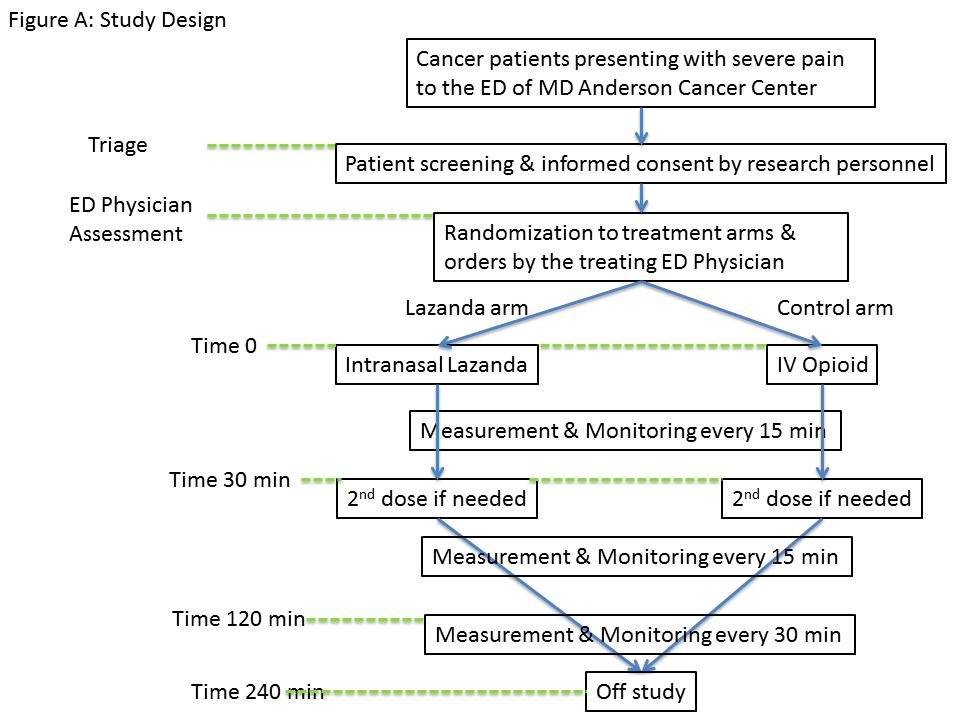


Figure A: **Schematic diagram of the study design.** The **primary objective** of this project is to test the non-inferiority of fentanyl nasal spray versus intravenous opioids in the change in pain intensity at one hour, starting from the time of drug delivery (time 0). The patient will be evaluated by the responsible ED physician with physical examination, serum chemistries, and other diagnostic evaluations as appropriate according to current standard practice. After signing the informed consent, the research nurse will log on to a clinical research administration website [http://www.oncologyresearch.org/ows-doc/index.htm (CORe)] and randomize the patient to the study arms. The time of randomization will be time stamped by the randomization software. The physician order for the study medication or the IV opioid will be signed by the ED physician.

**Baseline Measures**

The following baseline evaluations will be performed on consented subjects prior to study drug administration: vital signs, oxygen saturation, Ramsay Sedation Scale (Table 2) (Ramsay, 197).

The baseline pain intensity will be rated using NRS, and a blood sample will be drawn for standard of care laboratory investigation of the cause of pain or exacerbation of pain.

**Intervention:**

Intervention Arm: A Lazanda dose [100 mcg] at time 0 (defined as the time when intranasal Lazanda spray is administered) with a rescue dose allowed at time 0.5 hour (h).

Control Arm: An equipotent opioid dose [hydromorphone 1.5 mg pushed intravenously (IV)] at time 0 (defined as the time of completion of opioid IV push) with a rescue dose allowed at time 0.5 hour (h).

For subjects with pain NRS > 4 who desire additional analgesics at thirty minutes after Time 0 (i.e., Time 30 minutes), a second dose of medication will be administered as in the table above. Subjects with excessive sedation (Ramsay Sedation Scale < 4) will not receive analgesics at Time 30 minutes.

After another 90 minutes (i.e., Time 120 minutes), for patients with unrelieved pain (NRS >6 and Ramsay Sedation Scale <4), the patient may receive supplemental IV bolus doses of opioid as clinically indicated and ordered by the treating physician.

Table 2. **Ramsey Sedation Scale**

| Wakeful State | Scale | Description |
| --- | --- | --- |
| If Awake | Ramsey 1 | Anxious, agitated, restless |
|  | Ramsey 2 | Cooperative, oriented, tranquil |
|  | Ramsey 3 | Responsive to commands only |
| If Asleep | Ramsey 4 | Brisk response to light glabellar tap or loud auditory stimulus |
|  | Ramsey 5 | Sluggish response to light glabellar tap or loud auditory stimulus |
|  | Ramsey 6 | No response to light glabellar tap or loud auditory stimulus |

**10 Evaluation during Study**

We will perform measurements described in this section for 4 hours after initiation of the drug intervention while the patient is in the ED. The average length of stay for patients presenting with severe pain to our ED is currently 9 hours.

**Pain Measurements:** Pain measurement by NRS will be obtained at the time of randomization, time of treatment initiation, and every 15 min from administration of the first dose of pain medication up to Time 120 min and then every 30 min from Time 120 min to Time 240 min. At 60 min after randomization, one additional NRS rating will be obtained.

**Primary Outcome Measure:**

The primary outcome measure is the non-inferiority of fentanyl nasal spray versus IV opioids in the treatment-wise response starting from the time of drug delivery. The decrease in pain level between treatment initiation and one hour after will be compared between intranasal fentanyl and an analgesic equivalent dose of intravenous opioid.

**Secondary outcome measures**:

For secondary objective 1, we will compare the decrease in pain level between randomization and one hour after randomization between intranasal fentanyl and an analgesic equivalent dose of intravenous opioid.

For secondary objective 2, we will monitor the safety and side effects of fentanyl nasal spray and intravenous opioid. Safety evaluation will include vital signs, oxygen saturation and respiratory rate. Blood pressure, heart rate, oxygen saturation and respiratory rate will be monitored for abnormalities. Adverse reactions of nausea and vomiting and neuropsychiatric adverse effects will be monitored. Sedation will be measured by Richmond Agitation Sedation Scale (RASS). The side effects rating scale for dissociative anesthetics (SERSDA) will be assessed at the end of the study period.

For secondary objective 3, we will estimate the time from randomization to IV establishment in adults with cancer presenting to the ED with severe pain.

**Exploratory measures:**

Time to maximal pain relief and Summed Pain Intensity Difference (SPID) (Davies et al., 2011) will be derived using NRS data for the 4-hour period for additional exploratory analyses. Time to maximal pain relief is defined as the time duration from Time 0 to the first occurrence of the lowest NRS in a subject. Pain intensity difference (PID) at a time point is defined as the NRS at Time 0 minus the NRS at that time point. The PID over the 4-hour study period will be integrated as the SPID using the trapezoidal method to calculate the area under the curve.

**11 Adverse Events Recording**

***11.1. Adverse Events Monitoring:***

Adverse event monitoring and reporting will begin after patients receive the study drug or IV hydromorphone and will continue to be recorded through discharge from the study. Patients will be instructed to report all unusual events during the study. All adverse events and corresponding treatment will be recorded in the case report forms and summarized in the final clinical study report. Any ongoing adverse events will be followed up by phone if the patient is discharged from the hospital or by patient visit if the patient is hospitalized, within 24 hours after study completion.

Intranasal fentanyl is generally well tolerated with the most common side effects consisting of nausea, vomiting, headache, and sedation (side effects common to all opioids).

*Unexpected Adverse Drug Event*

All external adverse events/safety reports received from the sponsor will be submitted to the IRB through the Office of Protocol Research as described for the “External Adverse Event Report” located under section 1 of the OPR Forms Manual.

*Adherence to MDA Policy and Procedure for Reporting of Serious Adverse Events.*

This study will follow the MDA policy and procedures for reporting of adverse and serious adverse events. All adverse events and serious adverse events will be documented and available for review if desired. Criteria for reporting of events before routine review will be determined based on established institutional guidelines.

**12 Criteria for Evaluation and Endpoint Determination**

*Measurements*

NRS measures will be obtained at triage, randomization, time 0 (initiation of drug intervention) and every 15 minutes for 120 minutes and then every 30 minutes from time 2 to 4 hours, and at 60 min after randomization.

Vital signs, oxygen saturation and respiratory rate will be recorded at each time point of NRS measurement.

Adverse reactions of nausea and vomiting and neuropsychiatric adverse effects will be monitored.

Sedation will be measured by Richmond Agitation Sedation Scale (RASS) (Table 3) at Time 1 h (i.e., one hour after initiation of drug delivery). RASS is a scale used to grade the sedation or agitation level of a patient in a clinical setting (Sessler et al., 2002).

Table 3: **Richmond Agitation Sedation Scale**

| **Points** | **Classification** | **Description** |
| --- | --- | --- |
| 4 | Combative | Overtly combative or violent; immediate danger to staff |
| 3 | Very agitated | Pulls on or removes tube(s) or catheter(s) or has aggressive behavior toward staff |
| 2 | Agitated | Frequent nonpurposeful movement or patient–ventilator dyssynchrony |
| 1 | Restless | Anxious or apprehensive but movements not aggressive or vigorous |
| 0 | Alert and calm |  |
| -1 | Drowsy | Not fully alert, but has sustained (more than 10 seconds) awakening, with eye contact, to voice |
| -2 | Light sedation | Briefly (less than 10 seconds) awakens with eye contact to voice |
| -3 | Moderate sedation | Any movement (but no eye contact) to voice |
| -4 | Deep sedation | No response to voice, but any movement to physical stimulation |
| -5 | Unarousable | No response to voice or physical stimulation |

The side effects rating scale for dissociative anesthetics (SERSDA) (Table 4; Eide et al., 1994) will be assessed at time 4h.

Table 4. **Side effects rating scale for dissociative anesthetics**

|  | Severity Scale: 0, no change; 1, weak; 2, modest; 3, bothersome; 4, very bothersome |
| --- | --- |
| Side Effects | Severity |
| Fatigue |  |
| Dizziness |  |
| Headache |  |
| Feeling of unrealitiy |  |
| Changes in hearing |  |
| Changes in vision |  |
| Mood change |  |
| Generalized discomfort |  |
| Hallucination |  |
| SERSDA score = | Sum of the Severity Scores of the above side effects |

*Study Variables*

PROCEDURE-WISE ANALGESIC EFFICACY: The change in pain level between randomization and one hour after randomization will be compared between the intranasal fentanyl arm and the intravenous opioid arm. The power calculations are based on this primary efficacy variable.

TREATMENT-WISE ANALGESIC EFFICACY: Starting from the time of initiation of drug administration, the change in pain level after one hour will be compared between intranasal fentanyl arm and intravenous opioid arm.

TOLERABILITY: Tolerability to common adverse effects such as nausea and vomiting will be monitored. Neuropsychiatric adverse effects will be assessed by RASS and SERSDA. RASS score will be obtained at Time 1 h. SERSDA will be assessed after the final pain rating.

SAFETY: Blood pressure, heart rate, oxygen saturation and respiratory rate and adverse reactions will be monitored. Reports of adverse events will be collected spontaneously and in response to nondirected questioning. Investigators will ask how long the adverse effect lasted and categorize these events by seriousness (mild, moderate, or severe) and relationship to treatment (not related, doubtful, possible, probably, or very likely).

EMERGENCY CARE PROCESS CHARACTERIZATION: The time to IV establishment of adults with cancer presenting to the ED with severe pain after randomization will be estimated. Patients in both arms will have IV access. This time can be estimated using all the study participants. The time to IV establishment can also be compared between the 2 study arms to check for bias in work flow in this open label study.

**13 Data and Protocol Management**

All data from this study will be entered into a secured (password protected) database. Hard copy records of the consent and copies of the survey instruments will be stored securely and maintained by the study staff. Patient identifiers will be handled in a HIPAA-compliant manner. Confidentiality will be protected by identifying all medical information by the study with patient accession numbers, rather than storage by patient name or medical record number.

**14 Criteria for Removal from Study**

Since only one dose of Lazanda with one rescue dose in 30 min will be administered, patients with adverse reactions or side effects will not be removed from the study. The adverse reactions or side effects will be managed medically according to standard care.

**15 Statistical Considerations**

*Sample Size Calculations*

This is a two-arm study enrolling 84 cancer patients who present to the emergency department with severe pain (NRS pain score ≥ 7). Consented participants will be equally randomized to receive fentanyl nasal spray or IV hydromorphone for pain treatment. MD Anderson’s Clinical Oncology Research System (CORe) will be used to register participants and randomize participants to the study treatment arms.

The primary objective of the study is to examine the important secondary objective of testing the non-inferiority of nasal fentanyl relative to IV hydromorphone. At one hour after treatment *initiation* (rather than randomization) the change in NRS pain ratings for each group are assumed to be equal. We will have 80% power to detect non-inferiority of fentanyl nasal spray versus IV hydromorphone using a one-sided two-sample t-test, with a margin of equivalence of 0.9 and a significance level of 0.05 (Hintz, 2004). This sample size will also give us acceptable power to compare the two treatment groups on the pain intensity change from randomization (baseline) to one hour after randomization. Based on data presented in a meta-analysis of buccal and sublingual administration of fentanyl by Jandhyala et al. (2013), we estimate that the NRS pain intensity decrease from baseline will be 3.4 (SD 1.86) in the intranasal fentanyl arm at one hour after randomization. Although we are administering fentanyl using the nasal spray in the current study, the speed of absorption through the nasal mucosal should be similar to the speed of absorption through buccal or sublingual mucosa. Assuming that IV hydromorphone works at a speed similar to that of transmucosal fentanyl and that there is an average delay of 30 minutes from randomization to IV administration of hydromorphone, the expected NRS pain intensity decrease from baseline in the IV hydromorphone arm is 2.4 (SD 1.40) at one hour after randomization. A t-test with a 0.05 two-sided significance level will have 80% power to detect a 1-point difference in pain intensity decrease reported by the fentanyl group versus the IV hydromorphone group when the sample size is 84 (42 per group) using unequal variances by treatment group (Jandhyala et al., 2013). With an accrual rate of 8 participants per month, the expected enrollment duration is 10.5 months. Early withdrawal from the study is expected to be minimal and to be 5% at most. No adjustments to sample size will be made based on attrition.

*Statistical Analysis*

All participants randomized in the study (intention-to-treat population) will be included in the primary endpoint analysis. Should a participant withdraw from the study before the 1-hour NRS pain assessment, the NRS value used in the analysis will be the last value carried forward. In addition, we will conduct a sensitivity analysis that includes only participants with a 1-hour post randomization NRS pain rating (per-protocol population). Study dropout rates will be reported by treatment arm.

The mean change in pain intensity from treatment *initiation* until one hour after treatment initiation will be compared between the two arms. Non-inferiority will be evaluated by testing whether the lower bound of the 90% CI for difference in pain change scores is greater than -0.9, the margin of equivalence. Specifically, the mean change in NRS pain scores (assessed on an 11-point Likert scale with 0 = no pain and 10 = worst pain) from treatment initiation to one hour post-initiation will be calculated for both groups. The group difference in pain change scores (mean pain change in the fentanyl group minus that in the IV hydropmorphone group) and the associated two-sided 95% CI for the difference will be estimated. Non-inferiority of nasal fentanyl over IV hydromorphone will be concluded if the lower bound of the 90% CI around the estimated difference in pain score change lies above -0.9 points. This analysis will be in the intention-to-treat population but will be repeated in the per protocol population for sensitivity analysis. Both sets of results will be considered for evaluating the study objective.

The secondary efficacy endpoint is the change in NRS pain scores from randomization to one hour after randomization, which will be summarized using mean, standard deviation, minimum, median and maximum values. A two-sample t-test will be used to assess group difference in pain score change. In addition, analysis of covariance will be used to determine whether significant differences exist between the nasal fentanyl and IV hydromorphone groups in pain intensity scores at one hour post randomization with treatment group (fentanyl vs. IV hydromorphone) and baseline pain intensity included as independent variables. Models will be constructed to adjust for relevant baseline demographic and clinical factors, such as age, treatment dose, and disease type. Additional exploratory analyses will include time to maximal pain relief as well as summed pain intensity ratings for the entire observation period calculated as the sum of time-weighted pain intensity scores. Group differences in the summed pain intensity will be assessed using the area under the curve derived using the trapezoidal rule (Cappelleri et al., 2009).

Because study treatments are FDA approved for treatment of breakthrough pain in cancer patients, no formal safety monitoring rule will be established; however, all side effects and adverse events will be monitored continuously by the principle investigator. To address the secondary objective #2, the safety analysis will include data summaries of treatment side effects and adverse events by study group. Side effects will be tallied and reported by number and percent for the worst reported severity. Laboratory values and clinical measures will be summarized by treatment group using simple descriptive statistics, such as mean change from baseline value to the 4 hours after treatment initiation or end of treatment, whichever occurs first.

In addition, any delayed side effects that occur during the first 24 hours will be reported. This randomized trial will be monitored by the MD Anderson Data Safety Monitoring Board.

For secondary objective # 3, we will calculate the mean and corresponding 95% confidence interval for time from randomization to establishment of the IV. Each of the 84 study patients will be included, because it is standard institutional procedure for all patients admitted for pain to the ED to receive an IV. A sample size of 84 produces a 95% confidence interval equal to the sample mean plus or minus 0.217 when the estimated standard deviation is 1.00. Analyses will be performed using SAS version 9.4 (SAS Institute Inc., Cary, North Carolina, USA). P values <0.05 will be considered statistical significant.

**16 Data Safety Monitoring Board**

This randomized trial will be monitored by the MD Anderson Data Safety Monitoring Board (MDACC DSMB).

**17 References**

Borland M, Jacobs I, King B, O'Brien D. A randomized controlled trial comparing intranasal fentanyl to intravenous morphine for managing acute pain in children in the emergency department. Ann Emerg Med. 2007;49(3):335-40.

Breivik H, Cherny N, Collett B, et al. Cancer-related pain: a pan-European survey of prevalence, treatment and patient attitudes. Ann Oncol 2009;20:1420-1433.

Bruera E, Nam Kim H. Cancer Pain. JAMA 2003;290:2476-2479

Cappelleri JC, Bushmakin AG, Zlateva G, Sadosky A. Pain responder analysis: use of area under the curve to enhance interpretation of clinical trial results. Pain Practice. 2009. Sep-Oct; 9(5): 348-53.

Chang AK, Bijur PE, Gallagher EJ. Randomized clinical trial comparing the safety and efficacy of a hydromorphone titration protocol to usual care in the management of adult emergency department patients with acute severe pain. Ann Emerg Med. 2011;58(4):352-9.

Cordell W, Keene K, Filwa, V, et al. The high prevalence of pain in emergency medical care. Am J Emerg Med 2002;20;165-169.

Costantini M, Ripamonti C, Beccaro, M, et al. Prevalence distress, management and relief of pain during the last 3 months of cancer patients’ life. Results of an Italian mortality follow-back survey. Ann Oncol 2009; 20:729-35.

Davies A, Sitte T, Elsner F, et al. Consistency of efficacy, patient acceptability, and nasal tolerability of fentanyl pectin nasal spray compared with immediate-release morphine sulfate in breakthrough cancer pain. J Pain Symptom Manage 2011; 41: 358–366.

Eide PK, Jorum E, Stubhaug A, Bremnes J, Breivik H. Relief of post-herpetic neuralgia with the N-methyl-D-aspartic acid receptor antagonist ketamine: a double-blind, cross-over comparison with morphine and placebo. Pain 1994;58:347-354.Esteva, F. J., Moulder, S. L., Gonzalez-Angulo, et al. Phase I trial of exemestane in combination with metformin and rosiglitazone in nondiabetic obese postmenopausal women with hormone receptor-positive metastatic breast cancer. Cancer Chemother Pharmacol. 2013; 71:63-72.

Fine P. Breakthrough pain. In: Bruera E, Portenoy R, editors. Cancer Pain. New York: Cambridge University Press; 2003. p.408-12.

Fry M, Holdgate A. Nurse-initiated intravenous morphine in the emergency department: efficacy, rate of adverse events and impact on time to analgesia. Emerg Med. 2002;14(3):249–54.

Gahir KK, Ransom PA. Intranasal diamorphine integrated care pathway for paediatric analgesia in the accident and emergency department. Emerg Med J. 2006;23(12):959.

Hintze, J. (2004). NCSS and PASS. Number Cruncher Statistical Systems. Kaysville, Utah. [www.ncss.com](http://www.ncss.com).

Jandhyala R, Fullarton JR, Bennett MI. Efficacy of rapid-onset oral fentanyl formulations vs. oral morphine for cancer-related breakthrough pain: a meta-analysis of comparative trials. J Pain Symptom Manage. 2013; 46(4):573-80.

Kanner R. The scope of the problem. In: Portenoy R, Kanner R, editors. Pain management: theory and practice. Philadelphia: FA Davis; 1996. P. 40.

Kendall JM, Reeves BC, Latter VS; Nasal Diamorphine Trial Group. Multicentre randomized controlled trial of nasal diamorphine for analgesia in children and teenagers with clinical fractures. BMJ. 2001;322(7281):261–5. Comment in: BMJ. 2001;322(7298):1367–8. BMJ. 2001;322(7298):1368.

Portenoy R, Mathur R. Cancer Pain. In: Yeung SC, Escalante CP, Gagel RF, editors. Medical Care of Cancer Patients. Connecticut: People’s Medical Publishing House; 2009. p.60-71.

Rahman NH, DeSilva T. A randomized controlled trial of patient-controlled analgesia compared with boluses of analgesia for the control of acute traumatic pain in the emergency department. Emerg Med J. 2012; 43:951-957.

Ramsay M, Savege T, Simpson B, Goodwin R. Controlled sedation with alphaxalone-alphadolone. British Medical Journal 1974; 2: 656-59.)

Ritsema TS, Kelen GD, Pronovost PJ, Pham JC. The national trend in quality of emergency department pain management for long bone fractures. Acad Emerg Med. 2007;14(2):163–9.

Rupp T, Delaney K. Inadequate analgesia in emergency medicine. Ann Emerg Med 2004;43:494-503.

Sessler CN, Gosnell MS, Grap MJ, et al. The Richmond Agitation–Sedation Scale. Amer J Resp Critical Care Med, 2002; 166: 1338-1344. doi: 10.1164/rccm.2107138

Todd K. Emergency medicine and pain: A topography of influence. Ann Emerg Med. 2004;43:504-506.

Todd KH, Ducharme J, Choiniere M, et al. Pain in the emergency department: results of the pain and emergency medicine initiative (PEMI) multicenter study. J Pain. 2007;8(6):460–6.

Vainio A, Auvinen A. Prevalence of symptoms among patients with advanced cancer: an international collaborative study. J Pain Symptom Manage 1996;12:3-10.

Van Den Beuken-van Everdingen M, De Rijke J, Kessels A, et al. Prevalence of pain in patients with cancer: A systematic review of the past 40 years. Ann Oncol 2007;18:1437-1449.

Vissers, D., W. Stam, et al. (2010). "Efficacy of intranasal fentanyl spray versus other opioids for breakthrough pain in cancer." Curr Med Res Opin 26(5): 1037-1045.

Vu K, Busaidy N, Cabanillas ME, Konopleva M, Faderl S, Thomas DA, O'Brien S, Broglio K, Ensor J, Escalante C, Andreeff M, Kantarjian H, Lavis V, Yeung SC. (2012). A Randomized Controlled Trial of an Intensive Insulin Regimen in Patients With Hyperglycemic Acute Lymphoblastic Leukemia. Clin Lymphoma Myeloma Leuk. 12:355-62.

World Health Organization. Cancer pain relief with a guide to opioid availability. 2^nd^ ed. Geneva: World Health Organization; 1996.
