# Supplementary material for: Intranasal fentanyl spray versus intravenous opioids for the treatment of severe pain in patients with cancer in the emergency department setting: A randomized controlled trial
Source: PLoS One. 2020 Jul 10;15(7):e0235461. doi: 10.1371/journal.pone.0235461 (PMC7351205; doi:10.1371/journal.pone.0235461)
Supplement: S4 File — (PDF) [file pone.0235461.s006.pdf]

THE UNIVERSITY OF TEXAS
MD Anderson Cancer Center
Office of Protocol Research

Institutional Review Board (IRB)
Unit 1637
Phone 713-792-2933
Fax 713-794-4589

To: Sai-Ching J. Yeung 09/14/2015
From: Kara M. Seales
CC: Cristina Gonzales, Denise M. Langabeer, Tiffany D. Mitchell, Evanna L. Thompson, Jason M. Reed, Sapna R. Amin, Mariam Hanna

MDACC Protocol ID #: 2015-0086
Protocol Title: A randomized trial to compare fentanyl nasal spray with intravenous opioids to treat severe pain in cancer patients in the emergency department setting.
Version: 04

Subject: Activation and Distribution of Protocol 2015-0086

This study is now active and ready for patient accrual.

The Informed Consent(s) will be available in the Informed Consent Printer Database within 30 minutes.

In the event of any questions or concerns, please contact the sender of this message at (713) 792-2933.

Kara M. Seales 09/14/2015 03:49:55 PM
